# Treatment of rheumatoid arthritis in the Medicare Current Beneficiary Survey

**DOI:** 10.1186/ar4201

**Published:** 2013-03-18

**Authors:** Daniel H Solomon, Edward Yelin, Jeffrey N Katz, Bing Lu, Tamara Shaykevich, John Z Ayanian

**Affiliations:** 1 Division of Rheumatology, Immunology, and Allergy, Brigham and Women's Hospital, 75 Francis Street, Boston MA, 02115, USA; 2 Division of Pharmacoepidemiology, Brigham and Women's Hospital, 75 Francis Street, Boston MA, 02115, USA; 3 Rosalind Russell Medical Research Center for Arthritis, University of California, San Francisco, California 94143-0920, USA; 4 Department of Orthopedic Surgery, Brigham and Women's Hospital, 75 Francis Street, Boston MA, 02115, USA; 5 Division of General Internal Medicine, Brigham and Women's Hospital, 75 Francis Street, Boston MA, 02115, USA

## Abstract

**Introduction:**

Numerous studies across different health systems have documented that many patients with rheumatoid arthritis (RA) do not receive disease-modifying anti-rheumatic drugs (DMARDs). Relatively little is known about correlates of DMARD use and whether there are socioeconomic and demographic disparities. We examined DMARD use during 2001 to 2006 in the Medicare Current Beneficiary Survey (MCBS), a longitudinal US survey of randomly selected Medicare beneficiaries.

**Methods:**

Participants in MCBS with RA were included in the analyses, and DMARD use was based on an in-home assessment of all medications. Variables included as potential correlates of DMARD use in weighted regression models included race/ethnicity, insurance, income, education, rheumatology visit, region, age, gender, comorbidity index, and calendar year.

**Results:**

The cohort consisted of 509 MCBS participants with a diagnosis code for RA. Their median age was 70 years, 72% were female, and 24% saw a rheumatologist. Rates of DMARD use ranged from 37% among those <75 years of age to 25% of those age 75 to 84 and 4% of those age 85 and older. The multivariable adjusted predictors of DMARD use include: visit with a rheumatologist in the prior year (odds ratio, OR, 7.74, 95% CI, 5.37, 11.1) and older patient age (compared with <75 years, ages 75 to 84, OR 0.58, 95% CI 0.37, 0.92, and 85 and over, OR 0.09, 95% CI 0.02, 0.31). In those without a rheumatology visit, lower income and older age were associated with a significantly reduced probability of DMARD use; no association of DMARD use with income or age was observed for subjects seen by rheumatologists. Race and ethnicity were not significantly associated with receipt of DMARDs.

**Conclusions:**

Among individuals not seeing rheumatologists, lower income and older age were associated with a reduced probability of DMARD use.

## Introduction

Rheumatic disease experts and their professional societies widely embrace the importance of early and sustained use of disease-modifying antirheumatic drugs (DMARDs) for rheumatoid arthritis (RA) [1,2]. Despite this recommendation, several population-based and community-based studies demonstrate under-use of these agents. Several large studies of community-based practice suggest that rates of DMARD use may be no higher than 50% [3-5], with some evidence of recent improvement [6,7]. A more complete understanding of patterns of DMARD use could inform potential quality improvement interventions.

Studies that have assessed correlates of under-use have identified several important variables. A prior study within the Medicare population and another using data from health plans suggested that specific race/ethnicity groups, such as black race, was associated with underuse [4,6]. The largest study of this topic used data from Medicare Managed Care plans enrolled in the National Committee on Quality Assurance's Healthcare Effectiveness Data and Information Set (HEDIS) program [6]. This study found several patient characteristics associated with lower DMARD use, including black race, lower income, and older age. All studies that have examined the inclusion of a rheumatologist in a patient's care have found this factor to be the strongest predictor of DMARD use, with a 2- to 7-fold increase in prescribing among patients seeing a rheumatologist [4,5,7,8]. These findings are consistent across various countries.

Just as with other health care interventions, including total joint replacement, cardiac revascularization and organ transplant, vulnerable populations (people at high risk for disparities in health and health care delivery, typically including groups categorized by age, gender, race, ethnicity, geography, and disability status) appear to receive DMARDs less often than the general population [9-11]. DMARDs are recommended as the standard of care for RA and have been shown to improve function and reduce pain. Thus, differences in care can be described as treatment disparities, which have been defined as 'racial or ethnic differences in the quality of healthcare that are not due to access-related factors of clinical needs, preferences, and appropriateness of intervention' [12].

We examined potential disparities in DMARD use for RA in the Medicare Current Beneficiary Survey (MCBS), a large representative survey of Medicare beneficiaries in the US. The goal of these analyses was to identify potential demographic and socioeconomic disparities in DMARD use in the total cohort and within the sub-groups of individuals who did and did not see a rheumatologist.

## Methods

### Study cohort and design

The MCBS is conducted by the Center for Medicare and Medicaid Services on a representative sample of Medicare beneficiaries, excluding those exclusively in Medicare Advantage Plans (a Medicare health plan offered by a private company that contracts with Medicare to provide all physician and hospital benefits). Beneficiaries are visited in their homes and instructed to keep all paperwork related to medical services. Each year since 1992, approximately 4,000 to 6,000 Medicare beneficiaries have been recruited and then surveyed annually for four consecutive years. They assure its representativeness through a strategic sampling procedure. The MCBS sample is selected through a three-stage process that results in a sample of individuals who are more likely to live close to each other than a random draw of individuals. In the first stage, primary sampling units are selected consisting of metropolitan statistical areas or clusters of nonmetropolitan counties. In the second stage, ZIP code clusters are sampled within the primary sampling unit. In the third stage, beneficiaries are sampled within the ZIP code clusters. The MCBS also oversamples individuals under age 65 years (disabled) and age 85 or older (the oldest old) to increase the precision of estimates for these groups.

The survey includes sociodemographic information, medical illnesses, insurance information, region of residence, and medication use. There are few missing data in the MCBS survey, with one recent study finding 2.7% of all data elements missing [13]. In addition to the survey data from MCBS, we also had linked Part A and Part B health care claims from Medicare. This information includes ambulatory and inpatient diagnoses, visits and procedure codes. We used MCBS data from 2000 to 2006. Subjects entering in the last few years did not have complete follow-up over the four years of the survey; we included data from the available years through 2006.

Individuals with RA were required to meet two criteria: 1) report RA on the survey and 2) experience a health care claim coded with a diagnosis of RA. Those who were in a Medicare Advantage Plan must have had some period during which individual health care utilization claims were reported. We began follow-up of the study cohort at the first of these two criteria and continued through the end of the four years of surveys, allowing each participant to potentially contribute multiple years of follow-up. We required at least one year of follow-up after qualifying with a diagnosis of RA to determine subsequent DMARD use (the study outcome); thus, our last cases entered the cohort in 2005. Since several of our variables relied on data from the 12 months prior to qualifying for the definition of RA, our earliest cases entered the study cohort in 2001.

All of the MCBS data are publically available and do not require subjects' informed consent. Medicare performs the linkage and de-identifies the dataset. The Partners Healthcare Institutional Review Board approved the study protocol.

### DMARD use endpoint

DMARD use was based on an in-home assessment of all medications by MCBS interviewers. Participants were asked to retain all medication vials and prescription receipts. Detailed information on drug name, strength, and number of tablets was collected at the in-home visits. The medication data reported in the survey were assessed for report of any non-biologic (azathioprine, cyclosporine, d-penicillamine, hydroxychloroquine, leflunomide, methotrexate, and sulfasalazine) or biologic (abatacept, adalimumab, etanercept, infliximab, kineret, and rituximab) DMARDs used during the study time period. We also examined the medication reports for oral glucocorticoids, but these were not included in the DMARD category.

Participants were followed over several years and may have used DMARDs in certain years but not in others. Thus, each year of follow-up was considered a separate observation for the endpoint of DMARD use, allowing subjects to contribute multiple endpoints.

### Potential predictors of DMARD use

#### Sociodemographic

We defined several sociodemographic variables as potential predictors of DMARD use. These included age at the start of follow-up, gender, self-reported race/ethnicity, annual household income and educational attainment. Participants' residences were coded to US Census regions.

### Other variables

Several other variables of interest are available in MCBS and were assessed as potential predictors of DMARD use. We assessed three aspects of insurance status. All subjects had health care insurance through Medicare, but some also had supplemental private insurance. Some had drug insurance during the period prior to 2006, when all Medicare beneficiaries became eligible for Medicare Part D drug insurance. Finally, some noted being enrolled in a Medicare managed care plan. Other variables of interest included a visit to a rheumatologist based on provider types provided in Medicare claims data, the number of self-reported comorbid conditions [14] and the calendar year.

### Statistical analysis

We first examined the characteristics of all participants meeting the definition of RA, based on information collected for the year that they entered the RA cohort. Participants were then followed from the first survey year when they received a diagnosis code of RA through the end of their time in the MCBS, usually spanning one to three years. During each year, medication information was examined for the use of any DMARD as well as biologic DMARDs and oral glucocorticoids.

We then analyzed predictors of DMARD use in multivariate logistic regression models. All variables were considered a priori as potentially related to DMARD use and thus were included in the final models. Because of the repeated measures of DMARD use, each year of follow-up was considered as a separate observation with adjustment for within subject correlation using generalized estimating equations [15]. The primary model included all subjects with RA and all years of follow-up. Since seeing a rheumatologist was known to be an important factor in DMARD use, secondary models were developed that included only years of follow-up during which a rheumatologist was seen and the converse, models including only years of follow-up during which no rheumatologist was seen. Since we found that seeing a rheumatologist was an important predictor of DMARD use, we also constructed a multivariate logistic regression model that predicted seeing a rheumatologist as the dependent variable.

We used the MCBS sampling weights to account for the complex survey design and to calculate nationally representative estimates. Analyses were conducted using SAS 9.0 (SAS Institute Inc, Cary, NC, USA), except the weighted analyses which were conducted using SUDAAN statistical software version 10.0.1 (Research Triangle Institute, Durham, NC, USA).

## Results

From a total of 66,897 participants in MCBS during 2001 to 2006, we found 509 (0.76%) who fulfilled our definition of RA and were included in these analyses. The characteristics of the cohort are given in Table 1 and reflect the composition of Medicare beneficiaries with a median age of 70 years. As expected in an RA cohort, 72% were female. The majority was white and non-Hispanic and about one-third of the cohort had an annual income level ≤ $15,000. Private insurance in addition to Medicare was common (61.9%), few were in Medicare managed care (10.8%) and during this time period most (75.1%) had drug insurance. The average follow-up after entry into the RA cohort was 2.75 years.

**Table 1:** Characteristics of 509 subjects from the MCBS (2001 to 2006) included in the rheumatoid arthritis cohort

	N (%) or median (IQR)
Age at beginning of index round, years	70.1 (IQR 68.0 to 74.9)
Female	366 (71.9%)
Race/ethnicity	
Non-Hispanic, white	389 (76.4%)
Non-Hispanic, black	58 (11.4%)
Hispanic	44 (8.6%)
Non-Hispanic, Other	18 (3.5%)
Region (at index round)	
Northeast	108 (21.2%)
Midwest	120 (23.6%)
South	176 (34.6%)
West	96 (18.9%)
Missing	9 (1.8%)
Years of education at index round	
≤ high school	343 (67.4%)
Beyond high school	166 (32.6%)
Annual income (US dollars)	
>50,000	26 (5.1%)
>30,000 to 50,000	103 (20.2%)
>20,000 to 30,000	110 (21.6%)
>15,000 to 20,000	64 (12.6%)
≤ 15,000	187 (36.7%)
Missing	19 (3.7%)
Rheumatology visit during prior year	120 (23.6%)
Number of comorbid conditions during index round	
0 comorbid conditions	19 (3.7%)
1 comorbid condition	183 (36.0%)
2+ comorbid conditions	307 (60.3%)
Insurance status	
Private insurance	315 (61.9%)
Managed care	55 (10.8%)
Drug insurance	382 (75.1%)

All variables were measured at the survey round when individuals entered the rheumatoid arthritis study cohort. MCBS, Medicare Current Beneficiary Survey; IQR, interquartile range.

When examined year-by-year during follow-up, the percentage of subjects receiving any DMARD, a non-biologic DMARD or a biologic DMARD increased slightly over time (see Table 2). Non-biologic DMARDs use increased from 28% to 36% and biologic DMARD use increased from 2% to 7% during this time period (*P*-values for trend <0.05 for both). The percentage of persons using any oral glucocorticoids (alone or in combination) ranged from 28% to 37%. As well, a sizeable percentage (17% to 29%) used only oral glucocorticoids (without a DMARD).

**Table 2:** DMARDs and oral glucocorticoid use among subjects with rheumatoid arthritis in the MCBS, 2001 to 2006

	2001	2002	2003	2004	2005	2006
Number of patients	291	334	316	244	148	70
Any DMARDS	82 (28%)	83 (25%)	85 (27%)	88 (36%)	55(37%)	25 (36%)
Non-biologic DMARD	80 (27%)	82 (25%)	80 (25%)	85 (35%)	51 (35%)	22 (31%)
Methotrexate	49 (17%)	47 (14%)	42 (13%)	50 (20%)	31 (21%)	12 (17%)
Sulfasalazine	8 (3%)	5 (2%)	7 (2%)	5 (2%)	1 (0.5%)	1 (0.5%)
Hydroxychloroquine	25 (9%)	29 (9%)	29 (9%)	27 (11%)	15 (10%)	9 (13%)
Azathioprine	5 (2%)	3 (1%)	2 (1%)	3 (1%)	2 (1%)	0 (0%)
Leflunomide	6 (2%)	11 (3%)	12 (4%)	13 (5%)	8 (5%)	2 (3%)
Gold	0 (0%)	1 (0.3%)	2 (0.6%)	0 (0%)	0 (0%)	1 (1.4%)
Others	0 (0%)	0 (0%)	0 (0%)	1 (0.4%)	0 (0%)	0 (0%)
						
Biologic DMARD, any	6 (2%)	5 (2%)	11 (3%)	10 (4%)	14 (9%)	5 (7%)
Etanercept	3 (1%)	4 (1%)	8 (3%)	5 (2%)	6 (4%)	3 (4%)
Adalimumab	0 (0%)	0 (0%)	2 (1%)	3 (1%)	7 (5%)	2 (3%)
Infliximab	3 (1%)	1 (0.3%)	1 (0.3%)	2 (0.8%)	1 (07%)	0 (0%)
Anakinra	0 (0%)	1 (0.3%)	0 (0%)	0 (0%)	0 (0%)	0 (0%)
						
Oral steroid use, any	89 (31%)	94 (28%)	97 (31%)	82 (34%)	54(36%)	26 (37%)
Oral steroid use, only	50 (17%)	60 (18%)	60 (21%)	50 (18%)	41 (28%)	20 (29%)

Results are presented as number (%) of patients. Percentages are based on the number for a given year. They do not add up to 100% because the rows are not mutually exclusive categories. The sample size is greater than 509 because respondents were included in all years after they met the rheumatoid arthritis definition. However, the sample size decreased over time because of the requirement for at least 12 months of follow-up after a diagnosis of rheumatoid arthritis. DMARD, disease modifying anti-rheumatic drug; MCBS, Medicare Current Beneficiary Survey.

Table 3 demonstrates national estimates of DMARD use based on sample weights. During the study period, about two-thirds received any DMARD (non-biologic or biologic) at some point. However, less than 5% of those aged 85 and older received any DMARD, whereas almost a quarter of those in this age group received oral glucocorticoids. A smaller percentage of non-Hispanic black and Hispanic patients received any DMARD than white patients.

**Table 3:** DMARD and glucocorticoid use by patient characteristics among MCBS respondents with rheumatoid arthritis between 2001 and 2006, weighted to the US Medicare population

	Total weighted number (in thousands)	Any DMARD	Non-biologic DMARD	Biologic DMARD	Oral Glucocorticoid Use
		
		N (%)*			
Age, years					
65 to 74	2,965	1,089 (37%)	1,021 (35%)	169 (6%)	1,010 (34%)
75 to 84	1,338	328 (25%)	323 (24%)	31 (2%)	376 (28%)
85+	353	15 (4%)	15 (4%)	0	80 (23%)
Gender					
Female	3,440	1,071 (31%)	1,014 (30%)	173 (5%)	1,104 (32%)
Male	1,216	361 (30%)	346 (29%)	28 (2%)	361 (30%)
Race/ethnicity					
Non-Hispanic, white	2,996	1,170 (33%)	1,145 (32%)	130 (4%)	1,186 (33%)
Non-Hispanic, black	413	119 (25%)	111 (23%)	11 (2%)	141 (29%)
Hispanic	364	90 (20%)	52 (12%)	60 (13%)	117 (26%)
Non-Hispanic, other	133	52 (33%)	52 (33%)	0	22 (14%)
Income (US dollars)					
>50,000	260	126 (49%)	126 (49%)	30 (12%)	98 (38%)
>30,000 to 50.000	1,003	376 (38%)	353 (35%)	74 (7%)	353 (35%)
>20,000 to 30,000	992	309 (31%)	278 (28%)	65 (7%)	373 (38%)
>15,000 to 20,000	639	188 (30%)	175 (27%)	19 (3%)	175 (27%)
≤ 15,000	1,582	361 (23%)	358 (23%)	7 (0.5%)	392 (25%)

Total number = 4,656,213. *Percentage of the row total. DMARD, disease modifying anti-rheumatic drug use; MCBS, Medicare Current Beneficiary Survey.

The multivariable adjusted predictors of DMARD use are shown in Table 4. In the total cohort, the most influential predictor of DMARD use was a visit with a rheumatologist in the prior year (odds ratio, OR, 7.74, 95% CI, 5.37, 11.1). DMARD use declined significantly at older ages (compared with <75 years: ages 75 to 84, OR 0.58, 95% CI 0.37, 0.92, and age 85 and over, OR 0.09, 95% CI 0.02, 0.31).

**Table 4:** Regression models predicting DMARD use among respondents with rheumatoid arthritis in the MCBS, 2001 to 2006

	Total cohort	Among respondents without rheumatology visits	Among respondents with rheumatology visits
	**Odds ratio (95% confidence interval)**		

Number	1,403	1,058	345
Age, years			
65 to 74	1.00	1.00	1.00
75 to 84	0.59 (0.36, 0.95)	0.36 (0.17, 0.74)	0.95 (0.49, 1.84)
85+	0.10 (0.03, 0.33)	0.07 (0.01, 0.34)	0.13 (0.02, 0.91)
Gender, female	1.14 (0.69, 1.88)	0.99 (0.50, 1.94)	1.24 (0.63, 2.41)
Race			
Non-Hispanic, white	1.00	1.00	1.00
Non-Hispanic, black	0.61 (0.30, 1.22)	0.70 (0.23, 2.11)	0.63 (0.25, 1.59)
Hispanic	0.48 (0.18, 1.31)	0.39 (0.11, 1.38)	0.68 (0.18, 2.55)
Non-Hispanic, other	1.31 (0.53, 3.29)	1.55 (0.43, 5.57)	1.22 (0.26, 5.59)
Income (US dollars)			
>50,000	1.00	1.00	1.00
>30,000 to 50.000	0.76 (0.28, 2.08)	0.40 (0.14, 1.09)	1.12 (0.34, 3.67)
>20,000 to 30,000	0.57 (0.21, 1.57)	0.23 (0.08, 0.65)	1.06 (0.32, 3.57)
>15,000 to 20,000	0.59 (0.19, 1.81)	0.24 (0.08, 0.74)	1.01 (0.23, 4.51)
≤ 15,000	0.49 (0.18, 1.34)	0.16 (0.05, 0.48)	1.08 (0.31, 3.69)
Rheumatology care	7.69 (5.00, 11.1)	NA	NA

The sample size is greater than 509 because respondents were included in all years after they met the rheumatoid arthritis definition. Models were adjusted for variables in the table plus education, region of residence (South, Midwest, Northeast, and West), insurance status (private, managed care, and drug insurance), and number of comorbid conditions, as well as year of observation. These models were weighted based on the Medicare Current Beneficiary Survey (MCBS) sampling strategy. NA, not applicable; DMARD, disease modifying anti-rheumatic drug.

In analyses restricted to follow-up years associated without a rheumatologist visit, low income categories (<$30,000) were associated with significantly lower DMARD use. Significant differences in DMARD use by income were not observed among individuals who saw a rheumatologist in the current year, but those age 85 years and over were much less likely to report DMARD use. Race and ethnicity were not associated with DMARD use.

Since a rheumatologist visit was such a strong predictor of DMARD use, we examined predictors of a rheumatology visit (see Table 5). We found that older individuals were much less likely to see a rheumatologist (compared with age <75 years: ages 75 to 84, OR 0.59, 95% CI 0.39, 0.90, and over 85 years, OR 0.22, 95% CI 0.11, 0.48). As well, those with annual incomes less than $20,000 were less likely to see a rheumatologist, but gender, race, and ethnicity were not significant predictors of seeing a rheumatologist.

**Table 5:** Predictors of seeing a rheumatologist among 509 individuals with rheumatoid arthritis in the MCBS, 2001 to 2006

	Odds ratio (95% confidence interval)
Age, years	
65 to 74	1.00
75 to 84	0.59 (0.39, 0.90)
85+	0.22 (0.11, 0.48)
Gender, female	0.99 (0.70, 1.45)
Race	
Non-Hispanic, white	1.0
Non-Hispanic, black	1.15 (0.35, 3.79)
Hispanic	1.33 (0.44, 3.97)
Non-Hispanic, other	1.34 (0.49, 3.67)
Income (US dollars)	
>50,000	1.0
>30,000 to 50.000	0.55 (0.26, 1.16)
>20,000 to 30,000	0.47 (0.22, 1.01)
>15,000 to 20,000	0.32 (0.14, 0.74)
≤ 15,000	0.35 (0.16, 0.75)

Models adjusted for variables in the table plus education, region of residence (South, Midwest, Northeast, and West), insurance status (private, managed care, and drug insurance), and comorbidity index, as well as year of observation. MCBS, Medicare Current Beneficiary.

## Discussion

We studied DMARD use in a large nationally representative US cohort of older adult Medicare beneficiaries who self-reported RA and had been diagnosed as having RA by a health care provider. Similar to other community-based studies, we found suboptimal DMARD use among MCBS beneficiaries diagnosed with RA; approximately one-third of follow-up time in this study cohort demonstrated any DMARD use (see Table 2). Consistent with prior literature [4], a recent rheumatology visit was the strongest predictor of DMARD use. Older age was also significantly associated with a reduced probability of DMARD use in the total cohort. In addition, we found that those with low annual incomes were also significantly less likely to report DMARD use when they had not seen a rheumatologist. However, the income differences were not apparent in the groups seen by rheumatologists, but age 85 years and over remained associated with a reduced probability of DMARD use. In our primary analyses, race/ethnicity was not associated with a reduced probability of DMARD use.

First, we found that lower income was associated with a reduced probability of DMARD use among individuals not seeing a rheumatologist. This finding agrees with studies of treatment disparities in diabetes and hypertension [16,17] and is consistent with prior studies in RA [5,6]. Reduced DMARD use among those with lower annual income may be explained by difficulty affording medications or other unmeasured factors, such as reduced geographic access. The lack of DMARD among those with a lower income may also be related to patient attitudes, physician factors, or access issues.

Second, the reduced use of DMARDs in older patients has been found in several other analyses of RA [4,6,7]. Older individuals may have comorbidities that would be relative contraindications to DMARD use. We did control for a comorbidity index, but providers may perceive that certain diagnoses preclude the use of DMARDs. Moreover, it is possible that providers believe that RA is milder in older patients and thus less important to treat with DMARDs [18]. Some studies suggest elderly onset RA may be milder [18] and that older adults receive less aggressive treatment [19]. Another possibility is that more of the diagnosed RA in older adults is actually osteoarthritis; thus, there is differential misclassification by age explaining the DMARD finding.

Strengths of this study include the use of a large representative national sample as well as the breadth of potential predictors included in the MCBS. However, the findings must be interpreted in light of some important limitations. The definition of RA in the MCBS has not been validated. We required self-report and a physician diagnosis on a health care claim. Similar definitions have been used and found to be reasonably accurate [20], however there are very likely some individuals included in the RA cohort who do not have RA and may have other systemic rheumatic diseases (that is, polymyalgia rheumatica). This may partly explain the relatively low rates of DMARD use in our study. Differential misclassification of RA by patient characteristics could explain some of our findings. As well, average follow-up was 2.76 years; longer follow-up may have yielded different results. Furthermore, infusible DMARDs may not have been uniformly included in the in-home medication assessment. However, during the study period, only infliximab was given by infusion for RA. Another important limitation is that this study included only Medicare beneficiaries who are typically over 65 years of age and all have health insurance. This limits the generalizability of our findings but should not compromise internal validity. Additionally, when restricting the MCBS to RA patients, the sampling strategy may not yield a representative cohort of Medicare beneficiaries. Our dataset also contains no information about RA disease severity. Finally, it has been noted before that income is under-estimated in the MCBS [21].

## Conclusions

In conclusion, we found relatively low rates of DMARD use among individuals with a diagnosis code for RA in MCBS. Disparities in the use of DMARDs were pronounced among those without a rheumatology visit. In this group, older age and lower income were associated with a reduced likelihood of DMARD use. However, race/ethnicity did not predict DMARD use. Among those seeing a rheumatologist, older age remained significant but income was no longer associated with DMARD use. As in several other studies, seeing a rheumatologist is the strongest predictor of DMARD use. While this study did not focus on methods to improve quality of DMARD prescribing, our results suggest that quality improvement interventions focused on increasing appropriate DMARD use and improving access to rheumatologists are warranted. Another recent study found that a clinic specializing in care of medically vulnerable populations was able to reduce disparities in disease activity and function among persons with RA compared to a university tertiary care clinic [22]. This prior study and the present one suggest that disparities in utilization of suggested therapies and outcomes may be reduced by the way health care is organized, including appropriate referral to specialists and perhaps a special orientation to at-risk populations.

## Abbreviations

DMARD: disease-modifying antirheumatic drug; MCBS: Medicare Current Beneficiary Survey; OR: odds ratio; RA: rheumatoid arthritis.

## Competing interests

There are no direct competing interests to declare. Dr Solomon has received research grants from Abbott, Amgen and Lilly unrelated to the current work. He has also served in unpaid roles on two Pfizer trials not related to the current work.

## Authors' contributions

DHS designed the study, conducted the analyses, drafted the manuscript and received funding for the study. EY designed the study, conducted the analyses, and revised the manuscript. JNK designed the study, conducted the analyses, and revised the manuscript. TS analyzed the data and revised the manuscript. JZA designed the study, conducted the analyses, and revised the manuscript. All authors have read and approved the manuscript for publication.

## References

[B1] Saag KG Teng GG Patkar NM Anuntiyo J Finney C Curtis JR Paulus HE Mudano A Pisu M Elkins-Melton M Outman R Allison JJ Suarez Almazor M Bridges SL Jr Chatham WW Hochberg M MacLean C Mikuls T Moreland LW O'Dell J Turkiewicz AM Furst DE American College of Rheumatology 2008 recommendations for the use of nonbiologic and biologic disease-modifying antirheumatic drugs in rheumatoid arthritis Arthritis Rheum 2008 59 762 784 10.1002/art.23721 18512708

[B2] Smolen JS Landewe R Breedveld FC Dougados M Emery P Gaujoux-Viala C Gorter S Knevel R Nam J Schoels M Aletaha D Buch M Gossec L Huizinga T Bijlsma JW Burmester G Combe B Cutolo M Gabay C Gomez-Reino J Kouloumas M Kvien TK Martin-Mola E McInnes I Pavelka K van Riel P Scholte M Scott DL Sokka T Valesini G EULAR recommendations for the management of rheumatoid arthritis with synthetic and biological disease-modifying antirheumatic drugs Ann Rheum Dis 69 964 975 20444750 10.1136/ard.2009.126532 PMC2935329

[B3] Ward MM Fries JF Trends in antirheumatic medication use among patients with rheumatoid arthritis, 1981-1996 J Rheumatol 1998 25 408 416 9517756

[B4] Schmajuk G Schneeweiss S Katz JN Weinblatt ME Setoguchi S Avorn J Levin R Solomon DH Treatment of older adult patients diagnosed with rheumatoid arthritis: improved but not optimal Arthritis Rheum 2007 57 928 934 10.1002/art.22890 17665462

[B5] Lacaille D Anis AH Guh DP Esdaile JM Gaps in care for rheumatoid arthritis: a population study Arthritis Rheum 2005 53 241 248 10.1002/art.21077 15818655

[B6] Schmajuk G Trivedi AN Solomon DH Yelin E Trupin L Chakravarty EF Yazdany J Receipt of disease-modifying antirheumatic drugs among patients with rheumatoid arthritis in medicare managed care plans Jama 2011 305 480 486 10.1001/jama.2011.67 21285425 PMC3172813

[B7] Widdifield J Bernatsky S Paterson JM Thorne JC Cividino A Pope J Gunraj N Bombardier C Quality care in seniors with new-onset rheumatoid arthritis: a Canadian perspective Arthritis Care Res (Hoboken) 2010 63 53 57 20806274 10.1002/acr.20304

[B8] Shipton D Glazier RH Guan J Badley EM Effects of use of specialty services on disease-modifying antirheumatic drug use in the treatment of rheumatoid arthritis in an insured elderly population Med Care 2004 42 907 913 10.1097/01.mlr.0000135810.39691.f6 15319617

[B9] Mahomed NN Barrett J Katz JN Baron JA Wright J Losina E Epidemiology of total knee replacement in the United States Medicare population J Bone Joint Surg Am 2005 87 1222 1228 15930530 10.2106/JBJS.D.02546

[B10] Ayanian JZ Udvarhelyi IS Gatsonis CA Pashos CL Epstein AM Racial differences in the use of revascularization procedures after coronary angiography Jama 1993 269 2642 2646 10.1001/jama.1993.03500200056033 8487447

[B11] Kasiske BL Neylan JF Riggio RR Danovitch GM Kahana L Alexander SR White MG The effect of race on access and outcome in transplantation N Engl J Med 1991 324 302 307 10.1056/NEJM199101313240505 1898431

[B12] Williams RA Eliminating healthcare disparities in America: beyond the IOM report 2007 Totowa, NJ: Humana Press

[B13] Jonk Y O'Connor H Schult T Cutting A Feldman R Ripley DC Dowd B Using the Medicare Current Beneficiary Survey to conduct research on Medicare-eligible veterans J Rehabil Res Dev 47 797 813 21110253 10.1682/jrrd.2009.10.0174

[B14] Soumerai SB Pierre-Jacques M Zhang F Ross-Degnan D Adams AS Gurwitz J Adler G Safran DG Cost-related medication nonadherence among elderly and disabled medicare beneficiaries: a national survey 1 year before the medicare drug benefit Arch Intern Med 2006 166 1829 1835 10.1001/archinte.166.17.1829 17000938

[B15] Liang KY Zeger SL Longitudinal data analysis using generalized linear models Biometrika 1986 73 13 22 10.1093/biomet/73.1.13

[B16] Kanjilal S Gregg EW Cheng YJ Zhang P Nelson DE Mensah G Beckles GL Socioeconomic status and trends in disparities in 4 major risk factors for cardiovascular disease among US adults, 1971-2002 Arch Intern Med 2006 166 2348 2355 10.1001/archinte.166.21.2348 17130388

[B17] Booth GL Hux JE Relationship between avoidable hospitalizations for diabetes mellitus and income level Arch Intern Med 2003 163 101 106 10.1001/archinte.163.1.101 12523923

[B18] Deal CL Meenan RF Goldenberg DL Anderson JJ Sack B Pastan RS Cohen AS The clinical features of elderly-onset rheumatoid arthritis. A comparison with younger-onset disease of similar duration Arthritis Rheum 1985 28 987 994 10.1002/art.1780280905 4038365

[B19] Tutuncu Z Reed G Kremer J Kavanaugh A Do patients with older-onset rheumatoid arthritis receive less aggressive treatment? Ann Rheum Dis 2006 65 1226 1229 10.1136/ard.2005.051144 16414968 PMC1798297

[B20] MacLean C Park G Traina S Liu H Hahn B Paulus HE Khan K Positive predictive value of an administrative database algorithm for the identification of patients with rheumatoid arthritis [abstract] Arthritis Rheum 2001 44 S106

[B21] Poisal JA Reporting of drug expenditures in the MCBS Health Care Financ Rev 2003 25 23 36 15124375 PMC4194806

[B22] Barton JL Trupin L Schillinger D Gansky SA Tonner C Margaretten M Chernitskiy V Graf J Imboden J Yelin E Racial and ethnic disparities in disease activity and function among persons with rheumatoid arthritis from university-affiliated clinics Arthritis Care Res (Hoboken) 63 1238 1246 21671414 10.1002/acr.20525 PMC3169768

